# ﻿First discovery of troglobitic Paederinae (Coleoptera, Staphylinidae) from China

**DOI:** 10.3897/zookeys.1216.132155

**Published:** 2024-10-18

**Authors:** Cheng-Bin Wang, Li He

**Affiliations:** 1 Engineering Research Center for Forest and Grassland Disaster Prevention and Reduction, Mianyang Normal University, 166 Mianxing West Road, Mianyang 621000, Sichuan, China Mianyang Normal University Mianyang China; 2 State Grid Tianfu New Area Electric Power Supply Company, Chengdu 610094, Sichuan, China State Grid Tianfu New Area Electric Power Supply Company Chengdu China; 3 Sichuan Cave Exploration Team, No. 66, 5th Shuangcheng Road, Chenghua District, Chengdu 610051, Sichuan, China Sichuan Cave Exploration Team Chengdu China

**Keywords:** Cavernicolous, *Domene*, new species, Paederinae, rove beetle, Sichuan, subterranean, taxonomy

## Abstract

An unexpected troglobitic staphylinid is described from a dolomite cave in western China as *Domenelizeyui* Wang & He, **sp. nov.** (Coleoptera, Staphylinidae, Paederinae). The habitus of both sexes and important diagnostic features are illustrated. Brief notes on the habitat, biology and taxonomic status of the new species are provided. This is the first discovery of a troglobitic representative of Paederinae from China, the first record of a troglobitic *Domene* species, and only the third cavernicolous species of Paederinae from eastern Asia.

## ﻿Introduction

Currently in China, research on cave biodiversity is flourishing, and the first two textbooks on *Cave Biology* were published very recently (Liu 2021; Tian et al. 2023c). The last few decades have been particularly prolific in new findings of troglobitic beetles in China, for example Carabidae (Uéno and Wang 1991; Tian 2008; Tian and Clarke 2012; Deuve and Tian 2016; Tian et al. 2016, 2018, 2021, 2023a, 2023b; Chen et al. 2019; Tian and He 2020a, 2020b; Huang and Tian 2021; Jia et al. 2021), Cholevinae (Leiodidae) (Perreau and Růžička 2018), Pselaphinae (Staphylinidae) (Nomura and Wang 1991; Yin et al. 2010, 2011a, 2011b, 2015, 2016; Yin and Li 2015; Yin and Zhou 2018; Yin 2020; Yin and He 2020), and Dytiscidae (Spangler 1996; Wewalka et al. 2007; Zhao and Jia 2021). However, no obligate troglobites of Pselaphidae were known from China prior to this study—exclusive of troglophiles according to the screening criteria of Hlaváč et al. (2006); *Lathrobiumformidabile* Assing, 2013 from Sichuan of China is presumably a hypogean species, which was collected in a mixed forest, not in natural cave, probably by sifting leaf litter (Assing 2013).

Hlaváč et al. (2006) catalogued 44 species of troglobitic Staphylinidae worldwide, excluding the former Pselaphidae and Scaphidiidae. Among them, only four species are known from eastern Asia: two *Lathrobium* species (Paederinae) from Japan, one *Uenohadesina* species (Omaliinae) from South Korea, and one *Typhlomalota* species (Aleocharinae) from northern India.

For the genus *Domene* Fauvel, 1873 (Paederinae: Lathrobiini), 33 epigean species have been reported from eastern Asia (Koch 1939; Rougemont 1995; Assing and Feldmann 2014; Feldmann et al. 2014; Assing 2015, 2016, 2021; Peng et al. 2015, 2017; Schülke and Smetana 2015; Li 2019; Lin and Peng 2021): 24 species from Chinese mainland, two from Taiwan Island, five from Japan, two from Russia, two from Korea, two from Vietnam, and one from Myanmar; all belong to the subgenus Macromene Coiffait, 1982, except *D.hybrida* Assing, 2021, which was assigned to the monotypic subgenus Lobramene Assing, 2021 (Assing 2021).

In the present study, a fascinating troglobitic new species of *Domene* is described and illustrated from Taojindong [=Taojin Cave], a dolomite cave in Leshan Karst, Sichuan Province, western China. This species represents the first discovery of a troglobitic *Domene* species from eastern Asia. In addition, the problem of its taxonomic status is briefly discussed.

## ﻿Materials and methods

Specimens were relaxed and softened in an HH-2 digital homoeothermic water bath at 44.4 °C for 5 h and then placed in distilled water for cleaning and dissection. To examine the male genitalia, the abdomens after segments VII in morphological sense were detached using dissecting needles and cleared with a trypsin enzyme solution at room temperature for 12 h. They were then placed in 70% ethanol solution to remove the remaining trypsin. After examination, the dissected parts were stored in microvials with glycerin and attached below the respective specimens to which they belonged. Habitus images were taken using a Canon 50D DSLR with a Canon EF 100 mm f/2.8L IS USM lens and a dual LED fill light was used as the light source. Images of the morphological details were taken using a Canon macrophoto lens MP-E 65 mm on a Canon 5DsR. Images of the same object at different focal planes were combined using Zerene Stacker 1.04 stacking software. Adobe Photoshop CS6 was used for postprocessing. The terminology adopted in this paper for external features of the body and genitalia follows Lawrence et al. (2011).

The material examined for this study is deposited in the following collections: **CCZC**—collection of Chao Zhou, Chengdu, China; **CLHC**—collection of Li He, Chengdu, China; **CYLD**—collection of Yuan Li, Deyang, China; **CZWC**—collection of Zhen Wang, Chengdu, China; **CZYL**—collection of Ze-Yu Li, Panzhihua, China; **MYNU**—Invertebrate Collection of Mianyang Normal University, Mianyang, China; **SNUC**—Insect Collection of Shanghai Normal University, Shanghai, China.

Morphological measurements were taken using an ocular micrometer in millimetres (mm) of the following: **abdominal length**: length between the posterior margin of elytra and the abdominal apex along midline; **abdominal width**: widest part of abdomen; **antennal length**: length between the base and the apex of antenna; **body length**: length between the anterior margin of clypeus and the abdominal apex along midline; **elytral length**: length between the apex of scutellar shield and the posterior margin of elytra along suture; **elytral width**: widest part of both elytra combined; **eye length**: length of a single compound eye in lateral view; **forebody**: length between the anterior apex of clypeus and the posterior margin of elytra along midline; **head length**: length between the anterior margin of clypeus and the posterior constriction along midline; **head width**: widest part of head (including compound eyes); **neck region width**: widest part of neck region; **pronotal length**: length of the pronotum along midline; **pronotal width**: widest part of pronotum.

## ﻿Results

**Genus *Domene* Fauvel, 1873**

### ﻿ Domene lizeyui

Taxon classificationAnimaliaColeopteraStaphylinidae

Wang & He sp. nov.

22CBD7DF-B12D-541F-87D7-D526C5C91FBC

https://zoobank.org/0E20A8F4-460E-4C7B-9AAA-06C071B41AF3

**Figure 1.**
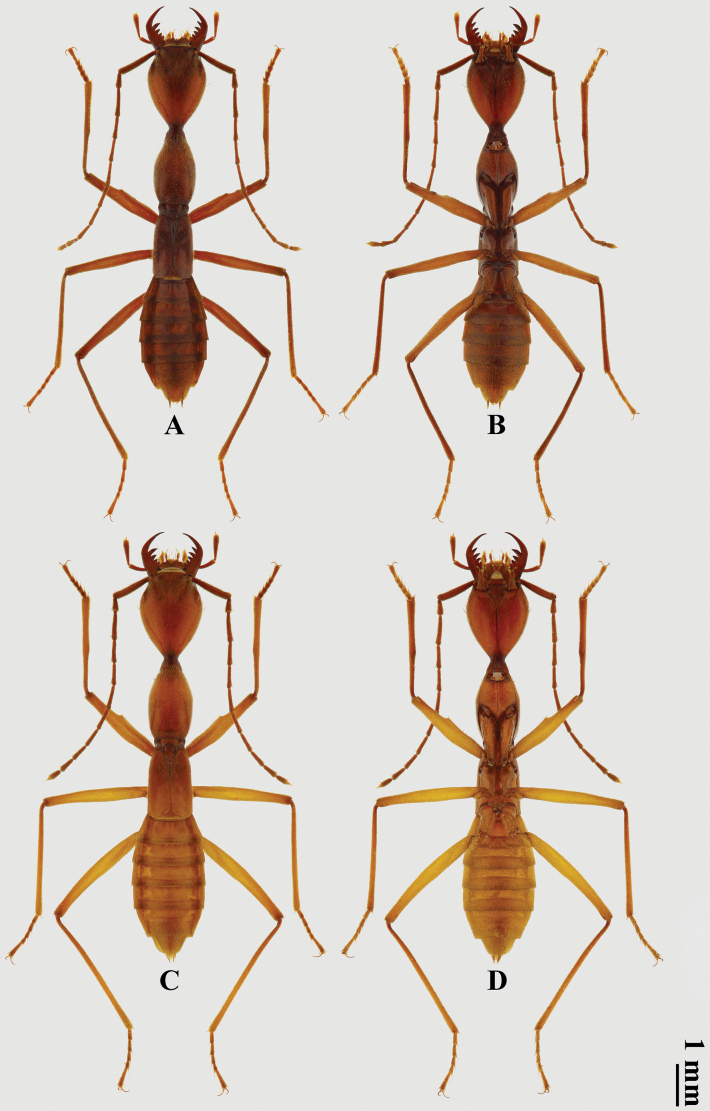
Habitus of *Domenelizeyui* Wang & He, sp. nov. **A, B** ♂, holotype **C, D** ♀, paratype (**A, C** dorsal views **B, D** ventral views).

**Figure 2.**
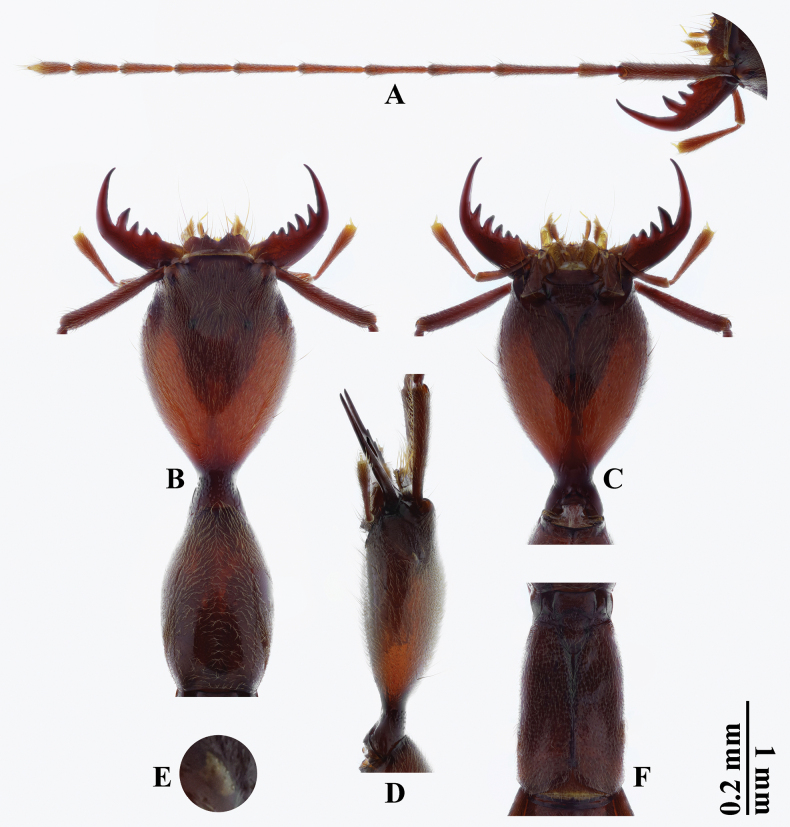
*Domenelizeyui* Wang & He, sp. nov., holotype, ♂ **A** left antenna **B** head and pronotum **C, D** head **E** left eye **F** elytra (**A, B, F** dorsal views **C** ventral view **D, E** lateral views). Scale bars: 1 mm (**A–D, F**); 0.2 mm (**E**).

**Figure 3.**
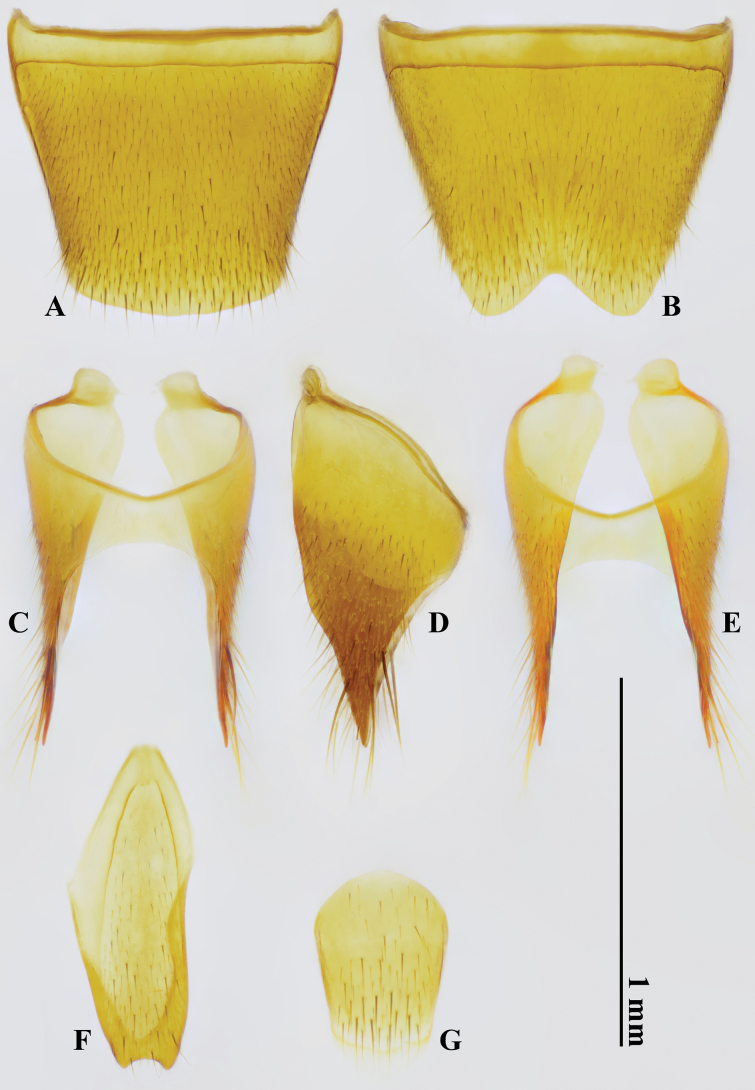
*Domenelizeyui* Wang & He, sp. nov., holotype, ♂ **A** tergite VIII **B** sternite VIII **C–E** tergite IX **F** sternite IX **G** tergite X (**A, C, G** dorsal views **B, E, F** ventral views **D** lateral view).

**Figure 4.**
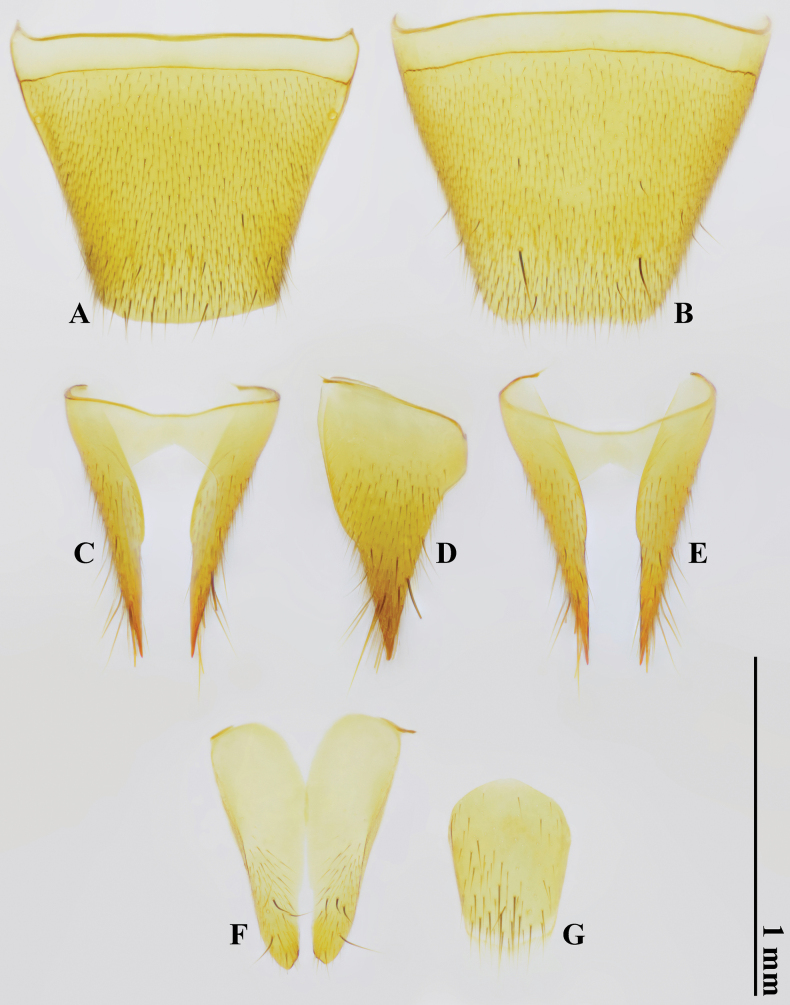
*Domenelizeyui* Wang & He, sp. nov., paratype, ♀ **A** tergite VIII **B** sternite VIII **C–E** tergite IX **F** sternite IX **G** tergite X (**A, C, G** dorsal views **B, E, F** ventral views **D** lateral view).

**Figure 5.**
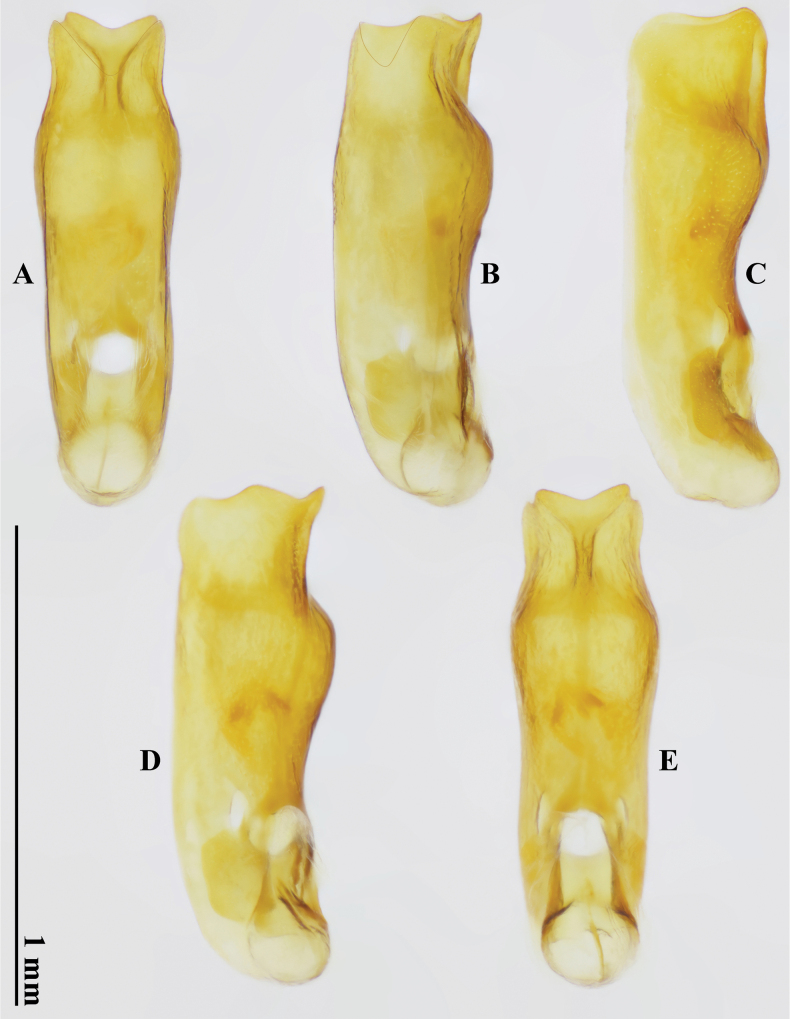
Aedeagus of *Domenelizeyui* Wang & He, sp. nov., holotype **A** ventral view **B** ventrolateral view **C** lateral view **D** dorsolateral view **E** dorsal view.

Figs 1 , 2 , 3 , 4 , 5 , 7

#### Type material.

***Holotype***: • ♂, China, Sichuan, Leshan City, Hulu Town, Shiqianggou, Taojindong [=Taojin Cave] [四川省乐山市沙湾区葫芦镇石墙沟淘金洞], 29.2965°N, 103.6370°E, alt. 513 m, 28.V.2023, Li He & Ze-Yu Li legg. (MYNU). ***Paratype***: • 3♂♂4♀♀. 3♀♀, same data as holotype (1♀ each in CLHC, MYNU and SNUC); • 3♂♂1♀, same data as holotype except 16.VI.2024, Yuan Li & Ze-Yu Li legg. (1♂ each in CCZC, CYLD and CZYL, 1♀ in CZWC).

#### Etymology.

The specific epithet is gratefully dedicated to one of the collectors of the type specimens, Mr Ze-Yu Li (Panzhihua, China), an enthusiastic amateur entomologist. The name is a noun in the genitive case.

#### Description.

**Male holotype. *Measurements*.** Body 8.5 mm long, widest at posterior angles of sternite V, 5.1 times as long as wide. Lengths of body parts: forebody 5.5 mm, head 2.0 mm, eye 0.1 mm, antenna 6.3 mm, pronotum 1.6 mm, elytra 1.2 mm, abdomen 3.0 mm; widths: head 1.4 mm, pronotum 1.0 mm, elytra 1.0 mm, abdomen 1.7 mm.

***Habitus*** (Fig. 1A, B). Body slender, with rather long and slender appendages, matt. Body almost entirely reddish brown; head with paired blackish paramedian spots at eye level; appendages with distal parts lighter in various degree. Body predominantly covered with short, recumbent, yellowish-brown pubescence.

***Head*** (Fig. 2B–D) oval, weakly convex dorsally, 1.4 times as long as wide, 1.4 times as wide as pronotum. Clypeus transverse, rather slightly emarginate at anterior margin; surface impunctate in anterior part. Fronto-clypeal suture absent. Frons distinctly concave. Antennal tubercles prominent, impunctate in apical parts. Surface irregularly, densely covered with fine punctures, attenuating posteriorly; interstices wider than diameter of punctures, lacking microsculpture. Eyes (Fig. 2E) extremely reduced, 0.06 times as long as postocular region in lateral view, lacking pigmentation, ommatidia unidentifiable. Neck region 3/8 width of head capsule. Gular sutures fused into longitudinally straight line, except at both ends.

***Mouthparts*** (Fig. 2B–D). Labrum transverse, deeply emarginate in middle of anterior margin and with paired subtriangular paramedian teeth. Mandibles sickle-shaped, large and strong, constantly curved and gradually tapered towards acute apices; left mandible with four inner teeth while right one with five. Maxillary palpi with four palpomeres, long and slender, with terminal palpomere rather thin and slender. Labial palpi with three palpomeres, slender, with terminal palpomere rather thin and slenderly coniform.

***Antennae*** (Fig. 2A) rather long and slender, 1.1 times as long as forebody and 4.5 times as long as head width. Antennomeres with length ratio from scape to antennomere 11 as follows: 2.5: 1.0: 1.9: 1.5: 1.4: 1.5: 1.5: 1.3: 1.2: 1.1: 1.0: 1.0. All antennomeres considerably longer than wide; scape longest, much thicker than other antennomeres; pedicel shortest; antennomere 3 second longest, 1.3 times as long as antennomere 4; antennomere 11 spindle-shaped, 3.2 times as long as wide.

***Pronotum*** (Fig. 2B) slenderly oblong, distinctly constricted anteriorly, weakly convex dorsally, 1.6 times as long as wide, widest around middle. Anterior margin rather narrow and arcuate; lateral margins from middle gradually narrowed posteriorly and distinctly so anteriorly; posterior angles roundly obtuse; posterior margin weakly emarginate. Dorsum with punctures similar to that of head but slightly finer; interstices without microsculpture; posterior half of median portion with shallow and impunctate sulcus.

***Scutellar shield*** (Fig. 2F) linguiform, rounded at apex. Surface with punctures coarser than that of head; interstices microreticulate.

***Elytra*** (Fig. 2F) long subtrapezoidal, 1.3 times as long as wide, widest at apicolateral angles, nearly as wide as and 0.8 times as long as pronotum, with apical parts distinctly apart. Lateral margins gradually divergent from humeri to apicolateral angles, then obliquely convergent to roundly obtuse apices. Dorsum slightly impressed on either side of scutellar shield and in middle portion, with punctures coarser than that of head; interstices microreticulate. Wings completely reduced.

***Legs*** rather long and slender. Coxae elongate. Femora slender, wider but shorter than tibiae. Tibiae thin, straight, each with two substraight and rather thin spurs at apex. Protarsi faintly widened; meso- and metatarsi slender; protarsi simple, not dilated; metatarsomeres 1–5 with length ratio as follows: 1.6: 1.7: 1.2: 1.0: 2.8. Claws rather thin, simply curved.

***Abdomen*** somewhat flattened dorsally, 1.8 times as long as wide, about half length of forebody, 1.7 times as wide as elytra, widest at posterior angles of sternite V. Tergites and sternites densely covered with fine punctures; interstices microreticulated. Tergites III–VII anteriorly with paired, ill-delimited, shallow impressions; sternites III–VII without modified setae. Tergite VII and sternite VII both unmodified, rather slightly emarginate at posterior margins.

***Male Terminalia and genitalia***. Tergite VIII (Fig. 3A) subtrapezoidal, without modified setae, simply curved at posterior margin; sternite VIII (Fig. 3B) without modified setae, deeply and subtriangularly excised at posterior margin. Tergite IX (Fig. 3C–E) unmodified; sternite IX (Fig. 3F) asymmetrical, longer than wide, and distinctly, roundly emarginate at posterior margin. Tergite X (Fig. 3G) shortly oblong, rounded at posterior margin. Aedeagus (Fig. 5A–E) large and symmetrical in ventral view, 1.0 mm long; posterior margin of ventral wall deeply and subtriangularly excised; ventral process absent.

**Male paratypes.** Body 8.2–8.6 mm long. Three male types without evident variations to the holotype.

**Female paratypes.** Body 8.9–9.2 mm long, similar to male in general appearance (Fig. 1C, D), but can be differentiated by the following characters: body generally larger; abdomen slightly slenderer, 1.9 times as long as wide; sternite VII with paired strong predistal setae; and the combination of following characters in terminalia and genitalia.

***Female terminalia and genitalia*.** Tergite VIII (Fig. 4A) subtrapezoidal, lacking modified setae, simply curved at posterior margin; sternite VIII (Fig. 4B) with paired strong predistal setae, truncated at posterior margin. Tergite IX (Fig. 4C–E) unmodified; sternite IX (Fig. 4F) bilobate, both slender, each with four strong predistal setae. Tergite X (Fig. 4G) shortly oblong, rounded at posterior margin.

#### Habitat.

**Figure 6.**
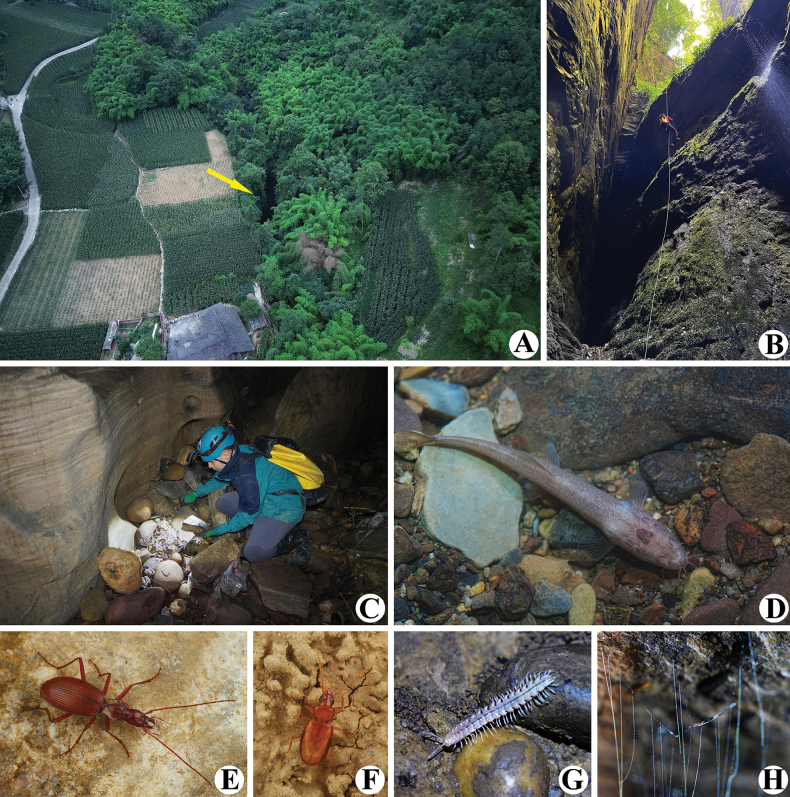
Taojindong [= Taojin Cave], the type locality of *Domenelizeyui* Wang & He, sp. nov. and some sympatric cave animals **A** environs of the cave (entrance shown by arrowhead) **B** Li He descending into the cave by using SRT **C** Ze-Yu Li collecting **D***Claea* sp. (Cypriniformes, Nemacheilidae) **E***Jujiroaduqianae* (Coleoptera, Carabidae) **F***Paratachys* sp. (Coleoptera, Carabidae) **G***Epanerchodus* sp. (Polydesmida, Polydesmidae) **H***Chetoneura* sp. (Diptera, Keroplatidae) (**A** © Xin-Yang Zou **B, D, G, H** © Ze-Yu Li).

The dolomite cave, Taojindong, is a shaft-type cave with a broad entrance located next to a backroad of Shiqianggou (Fig. 6A). Investigators can only descend into the cave by the single-rope technique (SRT) (Fig. 6B). During the rainy season, the water level in the cave rises, making access impossible; at other times, the cave is wet and the tunnel is spacious.

**Figure 7.**
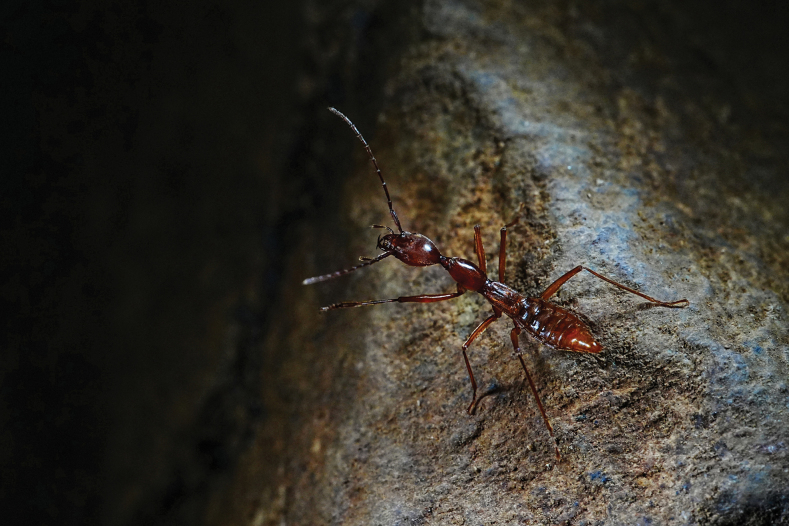
An individual of *Domenelizeyui* Wang & He, sp. nov. wandering on a rock in Taojindong (© Ze-Yu Li).

*Domenelizeyui* Wang & He, sp. nov. lives deep in the dark zone of the cave and was found either under rocks (Fig. 6C) or wandering on rock walls (Fig. 7; Suppl. material 1). Other troglobites found also inside the same cave were *Claea* sp. (Cypriniformes, Nemacheilidae) (Fig. 6D), *Jujiroaduqianae* Tian & He, 2023 (Coleoptera, Carabidae) (Fig. 6E), *Paratachys* sp. (Coleoptera, Carabidae) (Fig. 6F), *Epanerchodus* sp. (Polydesmida, Polydesmidae) (Fig. 6G), and *Chetoneura* sp. (Diptera, Keroplatidae) (Fig. 6H).

#### Differential diagnosis.

*Domenelizeyui* Wang & He, sp. nov. has no evident relatives to other cave congeners because it lives on the opposite side of Eurasia. It is readily distinguishable from its congeners in eastern Asia by its rather unique appearance, like integument depigmented, legs and antennae rather long and slender, eyes reduced, and wings absent. Moreover, it can be differentiated from its congeners by the combination of the following characters: labrum deeply emarginate in middle of anterior margin and with paired subtriangular paramedian teeth, abdomen shortened (about half length of forebody), modified setae absent on all tergites and sternites in male, and ventral process absent on aedeagus.

#### Distribution.

China (Sichuan).

## ﻿Discussion

The new species can be assigned to the genus *Domene* by the combination of the following characters (Coiffait 1982; Li 2009): body lacking bright-colored pattern; head dorsally weakly convex; eyes shorter than half length of temples; neck region wider than 1/5 width of head capsule; maxillary palpi with terminal palpomere thin and slender, smooth and glabrous; antennae slender; pronotum dorsally weakly convex, longer than wide; profemora each with a ventral protuberance around middle; tibiae lacking setae on lateral sides; protarsi faintly widened in both sexes; metatarsomere 5 shorter than length of basal four tarsomeres.

The discovery of the troglobitic *D.lizeyui* Wang & He, sp. nov. in China is of great interest for staphylinid taxonomy and biogeography. Species of the genus *Domene* were categorized into seven nominal subgenera (some species treated as incertae sedis) (Coiffait 1982; Oromí and Hernández 1986; Assing and Feldmann 2014; Assing 2018, 2021): *Domene* (s. s.) (Western Palaearctic), *Canariomene* Oromí & Hernández, 1986 (Canary Islands), *Lathromene* Koch, 1938 (Western Mediterranean), *Lobramene* Assing, 2021 (China), *Spelaeomene* Español, 1977 (Morocco), *Neodomene* Blackwelder, 1939 (Northern India; generic assignment doubtful), and *Macromene* Coiffait, 1982 (Eastern Palaearctic and Northern Oriental). As mentioned above, the new species is very different from known epigean species from eastern Asia in the subgenera *Lobramene* and *Macromene* by its striking troglomorphic features. Except *Spelaeomene*, other subgenera have their ventral processes on aedeagi more or less developed (absent in *Spelaeomene* and *D.lizeyui* Wang & He, sp. nov.). And the new species is easily distinguishable from *Spelaeomene* by its deeply, medially emarginate and subtriangularly, paramedianly toothed labrum on the anterior margin, and its short abdomen (about one-half length of forebody). Thus, it appears that a new subgenus needs to be established to accommodate this new species. However, as stated by Assing and Feldmann (2014: 499) “… the subgeneric concept currently in use is highly artificial. Taxa such as *Canariomene* and *Spelaeomene*, for instance, are mainly constituted by characters associated with adaptations to a hypogean habitat.” Therefore, we refrain from establishing a new subgenus at present because of the unavailability of specimens of other known troglobitic *Domene* species. A comprehensive phylogenetic analysis of the genus is urgently needed.

## Supplementary Material

XML Treatment for Domene lizeyui

## ﻿Additional information

### Author ORCIDs

Cheng-Bin Wang https://orcid.org/0000-0002-7913-8779

Li He https://orcid.org/0000-0002-4597-3442

### Data availability

All of the data that support the findings of this study are available in the main text or Supplementary Information.

## Supplementary materials

Supplementary material 1 A live *Domenelizeyui* Wang & He, sp. nov. wandering on rock walls (© Ze-Yu Li) Data type mp4 https://binary.pensoft.net/file/1156231This dataset is made available under the Open Database License (http://opendatacommons.org/licenses/odbl/1.0/). The Open Database License (ODbL) is a license agreement intended to allow users to freely share, modify, and use this Dataset while maintaining this same freedom for others, provided that the original source and author(s) are credited.Cheng-Bin Wang, Li He
